# Deep Q-Learning-Based Buffer-Aided Relay Selection for Reliable and Secure Communications in Two-Hop Wireless Relay Networks

**DOI:** 10.3390/s23104822

**Published:** 2023-05-17

**Authors:** Cheng Zhang, Xuening Liao, Zhenqiang Wu, Guoyong Qiu, Zitong Chen, Zhiliang Yu

**Affiliations:** 1 Key Laboratory of Modern Teaching Technology, Ministry of Education, Xi’an 710062, China; zhang_cheng@snnu.edu.cn (C.Z.); zqiangwu@snnu.edu.cn (Z.W.); qgyqgy@snnu.edu.cn (G.Q.); 2 School of Computer Science, Shaanxi Normal University, Xi’an 710119, China; zitong_@snnu.edu.cn; 3 Shaanxi Key Laboratory for Network Computing and Security Technology, Xi’an 710048, China; 4 School of Mathematics and Computer Science, Shaanxi University of Technology, Hanzhong 723001, China; yu@snut.edu.cn

**Keywords:** physical-layer security, buffer-aided relay selection, Markov decision process, deep Q-learning, secrecy outage probability, connection outage probability

## Abstract

This paper investigates the problem of buffer-aided relay selection to achieve reliable and secure communications in a two-hop amplify-and-forward (AF) network with an eavesdropper. Due to the fading of wireless signals and the broadcast nature of wireless channels, transmitted signals over the network may be undecodable at the receiver end or have been eavesdropped by eavesdroppers. Most available buffer-aided relay selection schemes consider either reliability or security issues in wireless communications; rarely is work conducted on both reliability and security issues. This paper proposes a buffer-aided relay selection scheme based on deep Q-learning (DQL) that considers both reliability and security. By conducting Monte Carlo simulations, we then verify the reliability and security performances of the proposed scheme in terms of the connection outage probability (COP) and secrecy outage probability (SOP), respectively. The simulation results show that two-hop wireless relay network can achieve reliable and secure communications by using our proposed scheme. We also performed comparison experiments between our proposed scheme and two benchmark schemes. The comparison results indicate that our proposed scheme outperforms the max-ratio scheme in terms of the SOP.

## 1. Introduction

With the development of 5G and beyond, wireless networks are widely used in various fields, such as wireless sensor networks (WSNs) [1], cognitive radio networks (CRNs) [2], and the Internet of Things (IoTs) [3]. With the wide use of wireless networks, a large amount of confidential information is transmitted over each network every day. However, signals may be undecodable at the receiver end due to the fading of wireless signals, and may be intercepted by an eavesdropper due to the broadcast nature of wireless channels, leading to critical reliability and especially security issues in wireless networks. Any unauthorized attacker within the transmission range of a transmitter can receive the transmitted information, which can easily cause information leakage [4]. Therefore, the problem of reliable and secure communications in wireless networks urgently needs to be solved.

The traditional method to achieve secure communications in wireless networks is based on a cryptographic mechanism. The principle of cryptography is to encrypt confidential information with a secret key at the legitimate sender’s end and then decrypt it with a secret key at the legitimate receiver’s end [5]. As the secret key is deployed only on the legitimate transmitter and receiver, eavesdroppers cannot decrypt the encrypted information because of the lack of the secret key [6]. The disadvantage of cryptography is that its implementation requires the deployment of devices with a high level of computational performance due to the high level of computational complexity associated with encrypting and decrypting. It is impossible to require all devices connected to wireless networks to have high computational ability. In recent years, a new method called physical-layer security (PLS), which has low computational complexity, has been proposed and is used to aid cryptography in achieving secure communications in wireless networks with low computing capability. The principle of PLS is based on information theory, and uses the randomness of noise and wireless channels to achieve secure communications [7]. Compared with the cryptography method, PLS has a lower network resource overhead and computational complexity [8]. Therefore, PLS has become a promising technique that can help in enhancing security performance in wireless networks.

Common PLS techniques include beamforming [9], artificial noise [10], and relay selection [11]. The principle of beamforming is to achieve the directional transmission of signals by adjusting the transmission direction of the antennas to achieve PLS [12]. Artificial noise interferes with eavesdropping by sending noise [13]. Relay selection achieves PLS by selecting the appropriate relay nodes to which to transmit confidential information [14]. Compared with beamforming and artificial noise, the implementation complexity of relay selection is lower. Depending on whether the relay nodes are equipped with buffers or not, the relay selection technique is divided into conventional and buffer-aided relay selection [15]. In conventional relay selection, once relay nodes without buffers receive the signals, they have to immediately forward them to the next hop [16]. In contrast, buffer-aided relay selection can temporarily store the received signals in buffers instead of transmitting them immediately [17]. So, buffer-aided relay selection can achieve better security performance than that of the conventional relay selection without buffers, especially when the channel quality is poor [18]. Due to its low implementation complexity and good security performance, we used the buffer-aided relay selection technique to achieve reliable and secure communications in this paper.

Traditional buffer-aided relay selection selects the best relay by adopting a central node that collects the network information (e.g., the channel state information (CSI) of the legitimate link and the CSI of the eavesdropping links) and then selects the relays online on the basis of this information. However, it is difficult to achieve CSI of eavesdropping links, as the eavesdroppers always transmit no information, and too much energy, storage, and time are needed to conduct the relay selection, as there are several transmission patterns (i.e., source–relay, relay–destination, and source–destination transmissions) and many possible relay buffer states during the transmission. This is challenged when the central node is resource-limited. Unlike traditional methods, traditional Q-learning (TQL) and DQL define the Q-function, which can simplify the modeling of information transmission in buffer-aided relay selection by evaluating the gain of choosing a particular link to which to transmit signals in the current state in an integrated manner, especially DQL. By using neural networks to fit the Q-function, DQL can create the Q-function without storing it in a Q table, reducing both spatial and temporal complexity for buffer-aided relay selection. Therefore, we used the DQL method to propose a new buffer-aided relay scheme to achieve reliable and secure communications in two-hop wireless relay networks.

## 2. Related Work

For two-hop buffer-aided relay networks without eavesdroppers, the authors in [19] consider the reliability of wireless communications and proposed a novel buffer-aided relay selection scheme called the max-link scheme. In the max-link scheme, the signals at each hop are transmitted by the link with the maximum signal-to-noise ratio (SNR) to achieve reliable communications. The authors in [19] also established a theoretical analysis framework on the basis of a Markov chain (MC) for analyzing the outage performance of their proposed buffer-aided relay selection scheme. The authors in [20] combine social networks with two-hop wireless relay networks and investigate how to design a buffer-aided relay selection scheme to achieve reliable communications when there are untrusted relays in the network. Due to the introduction of buffers, the queuing delay of data packets at buffer-aided relay increases. To achieve reliable communication and reduce delay, the authors in [21] proposed a delay-sensitive buffer-aided relay selection based on channel-based greedy scheduling in vehicular networks. Because of the low implementation complexity of buffer-aided relay selection, the authors in [22] use buffer-aided relay selection to improve the reliability of bidirectional wireless sensor network communications. These buffer-aided relay selection schemes described above are all based on MP. The authors in [23] model buffer-aided relay selection as a Markov decision process (MDP) rather than the MP and exploit TQL to design a buffer-aided relay scheme. TQL evaluates all links by Q-function and selects the link with the maximum Q-function each time to transmit the signals and thus achieves reliable communications. Due to the excellent reliability performance of the proposed scheme based on TQL, the authors in [24,25] extend this work to vehicular networks, D2D communications and achieve reliable communications in vehicular networks and D2D communications. Although these TQL-based schemes can achieve reliable communications, too much storage space is needed to store the Q-table and a high time cost to look up the Q-function in the Q-table as all Q-functions have to be stored in the Q-table.

As research continued, researchers began to investigate how to achieve secure communications using buffer-aided relay selection when considering possible eavesdroppers in the network [26,27,28,29,30,31,32]. Based on the work in [19], the authors in [26] consider the case where a passive eavesdropper is present and proposed a new buffer-aided relay selection scheme to achieve secure communications by selecting the link with the maximum instantaneous secrecy capacity at each hop to transmit the signals. In real scenarios, not only illegal eavesdroppers eavesdrop signals, but also untrusted relay nodes can intercept the transmitted signals as well. These untrusted relay nodes are both cooperators and potential eavesdroppers of information transmission. In response to the presence of untrusted relay nodes, the authors in [27] propose a secure buffer-aided relay selection scheme that uses the AF mode to avoid the decoding of confidential information by untrusted relay nodes. The authors in [28] extend this work to a more general scenario where both potential eavesdropping nodes and passive eavesdroppers are present. The authors in [29] extend secure communications to bidirectional wireless relay network and design a buffer-aided relay selection scheme based on an achievable rate. In addition, to resist the eavesdroppers, buffer-aided relay selection is often combined with full duplex (FD) [30], cooperative jamming (CJ) [31] and energy harvesting (EH) [32] to achieve secure communications. Although these schemes can realize secure communications, they also increase the implementation complexity, which conflicts with the original intent of adopting buffer-aided relay selection.

All of the above related works only consider the reliability or the security performances of wireless communications, without considering both the security and reliability issues. In fact, it is very challenging to simultaneously achieve reliable and secure communications by using buffer-aided relay selection. It requires simultaneously taking into account the legitimate channel quality, eavesdropping channel quality, buffer queues, secrecy rate, etc. Therefore, buffer-aided relay selection based on traditional methods is difficult to achieve reliable and secure communications while maintaining low implementation complexity. With the development of deep learning (DL), DL has been applied to wireless relay networks [33,34,35]. A large number of researchers have started to use deep learning to study buffer-aided relay selection [36,37,38,39,40,41,42,43]. The authors in [36] model the buffer-aided relay selection as a multi-classification problem and uses a deep neural network (DNN) to predict the suitable link to transmit the signals. Inspired by [23], the authors in [37] utilize DQL to solve the buffer-aided relay selection problem, where a modified version of TQL is used. Different from TQL, DQL uses DNN to fit the Q-function instead of storing Q-function in the Q-table. Therefore, DQL has lower time complexity and space complexity compared to TQL [38]. The comparison experiments in [39] demonstrated that DQL has better learning results and lower complexity than those of TQL, and the implemented scheme via DQL is more suitable for practical scenarios, as the implemented scheme via DQL could work without prior information. On this basis, the authors in [40,41] realized reliable communications for IoTs [40] and CRNs [41] by using the DQL-based buffer-aided relay selection schemes. The authors in [40,41] extend their work further and use the proposed DQL-based buffer-aided relay selection scheme to realize reliable and secure communications in CRNs [42,43].

DQL makes it possible to achieve reliable and secure communications using buffer-aided relay selection. However, the works in [42,43] use DQL to address the issue of power allocation (PA) to achieve reliable and secure communications and they did not consider possible eavesdroppers in the network. Therefore, this paper explores how to achieve reliable and secure communications using only a DQL-based buffer-aided relay selection scheme in the more common two-hop wireless relay networks rather than CRNs. To highlight the contributions of this paper, we give a comparison of our work with related works in Table 1. The work of this paper is summarized as follows:

- To propose a DQL-based buffer-aided relay selection scheme, we first analyze the communication model of a two-hop AF buffer-aided relay network with the presence of a passive eavesdropper and then model the information transmission process as an MDP.
- We then propose a DQL-based buffer-aided relay selection scheme to optimize the above MDP. In the proposed scheme, we consider both the legal channel states and eavesdropping channel states, buffer states, target rate and target secrecy rate and use DNNs to fit the Q-function and select the link with the maximum Q-function value each time.
- Finally, we verify the reliability and security performances of the proposed scheme by using Monte Carlo simulations. The reliability and security performances are measured by the COP and the SOP, respectively. Simulation results demonstrate that the proposed scheme can achieve reliable and secure communications. We also compare the COP and SOP of the proposed scheme with the max-link and max-ratio schemes, respectively. The comparison results show that the proposed scheme outperforms max-ratio schemes in terms of security performance.

The remainder of this paper is organized as follows: Section 3 introduces the system model; Section 4 introduces the framework of information transmission based on MDP; Section 5 describes the proposed buffer-aided relay selection scheme; Section 6 shows the simulation results of proposed scheme; Section 7 concludes the contributions of this paper.

## 3. System Model

**Figure 1:**
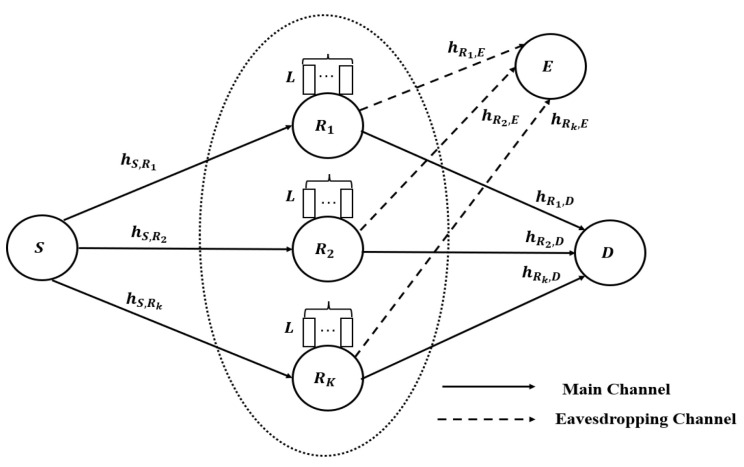
Illustration of the system model.

As depicted in Figure 1, this paper considers a two-hop AF buffer-aided relay network, which is composed of a source node *S*, a cluster of AF buffer-aided relay nodes $R_{k}$ (k∈{1,2,⋯,K}), a destination node *D* and a passive eavesdropper node *E*. The source node *S* cannot communicate with the destination node *D* directly due to path loss and the long distance, so the signals from *S* must be forwarded by the buffer-aided relay node $R_{k}$. The number of AF buffer-aided relay nodes is *K*. Every relay node is in half-duplex (HD) mode and is equipped with a buffer queue $Q_{k}$ of length *L*, so these relay nodes can store the received signals instead of forwarding them immediately to *D*. This paper assumes that the eavesdropping node *E* only eavesdrops the signals from $R_{k}$ to *D*, and does not eavesdrop the signals from *S* to $R_{k}$.

Without the decoding process at relays, AF relays can thus decrease the probability of being intercepted by potential eavesdroppers for transmitted signals [44]. Thus, we assume that all relays are AF relays in this paper to enhance the security of signals transmitted in the network.

We assume that all channels are independent and non-identically distributed quasi-static Rayleigh fading channels, including eavesdropping channels. In this paper, we use $h_{m,n}$ and $g_{m,n}$ to denote the channel coefficient and the channel gain between node *m* and node *n*, respectively, where $g_{m,n}={\left|h_{m,n}\right|}^{2}$. Since all channels are Rayleigh channels [45], the channel gain follows the exponential distribution, which means that $E[|h_{m,n}{|}^{2}]=E\left[g_{m,n}\right]=\Omega_{m,n}$, where E[.] is the expectation operator and $\Omega_{m,n}$ is the average channel gain. This paper assumes that the real-time CSI is completely known and sets the source node *S* as the central node, which receives the real-time CSI of all channels and buffers state information of all buffer-aided relay nodes then selects an appropriate link to transmit the signals according to relay selection schemes. Supposing at a time slot *t*, the central node selects an *S* to $R_{k}$ link to transmit signals, the received signals $y_{R_{k}}\left(t\right)$ at $R_{k}$ can be expressed as

(1) $$\begin{array}{c} y_{R_{k}}\left(t\right)=\sqrt{P_{s}}h_{S,R_{k}}\left(t\right)x_{s}\left(t\right)+n_{R_{k}}\left(t\right), \end{array}$$

where $P_{s}$ is the transmission power of the source node *S*, $x_{s}\left(t\right)$ is the signal sent by *S* at time *t*, and $n_{R_{k}}\left(t\right)$ is the additive white Gaussian noise (AWGN) noise with variance power $\sigma^{2}$ at $R_{k}$. According to (1), the instantaneous SNR of *S* to $R_{k}$ link at time *t* is given by

(2) $$\begin{array}{c} \psi_{S,R_{k}}\left(t\right)=\frac{P_{s}{\left|h_{S,R_{k}}\left(t\right)\right|}^{2}}{\sigma^{2}},k\in \left\{1,2,\cdots ,K\right\}, \end{array}$$

and the channel capacity of *S* to $R_{k}$ link is $C_{S,R_{k}}\left(t\right)=\frac{1}{2}\log_{2}\left(1+\psi_{S,R_{k}}\left(t\right)\right),\;k\in \left\{1,2,\cdots ,K\right\}$. The received signal $y_{R_{k}}\left(t\right)$ is stored in the corresponding buffer queue $Q_{k}$ waiting for the transmission to the next hop. After waiting for $t_{1}$ time slots, the received signal $y_{R_{k}}\left(t\right)$ is amplified to resist path fading and then forwarded to the destination node *D* by the buffer-aided relay node $R_{k}$. Thus, at time slot $t^{\prime }=t+t_{1}$, the signal $x_{R_{k}}\left(t^{\prime }\right)$ sent by the buffer-aided relay node $R_{k}$ is represented as

(3) $$\begin{array}{c} x_{R_{k}}\left(t^{\prime }\right)=A_{R_{k}}\left(t^{\prime }\right)y_{R_{k}}\left(t\right), \end{array}$$

where

(4) $$\begin{array}{c} A_{R_{k}}\left(t^{\prime }\right)=\frac{1}{\sqrt{P_{s}{\left|h_{S,R_{k}}\left(t\right)\right|}^{2}+\sigma^{2}}} \end{array}$$

is the amplification factor of the buffer-aided relay node $R_{k}$ at time $t^{\prime }$, it is determined by the quality of the channel between source node *S* and the buffer-aided relay node $R_{k}$ at time *t*. Due to the broadcast nature of wireless channel, eavesdropping nodes within the transmission range can also receive the transmitted signals. In this paper, we assume that the eavesdropping node only eavesdrops the signals sent by the buffer-aided relay nodes $R_{k}$ to the destination node *D*. So the signals received by *S* and *E* can be expressed as

(5) $$\begin{array}{c} y_{D}\left(t^{\prime }\right)=\sqrt{P_{R_{k}}}h_{R_{k},D}\left(t^{\prime }\right)x_{R_{k}}\left(t^{\prime }\right)+n_{D}\left(t^{\prime }\right),\\ y_{E}\left(t^{\prime }\right)=\sqrt{P_{R_{k}}}h_{R_{k},E}\left(t^{\prime }\right)x_{R_{k}}\left(t^{\prime }\right)+n_{E}\left(t^{\prime }\right), \end{array}$$

respectively, where $P_{R_{k}}$ is the transmission power of $R_{k}$, $n_{D}\left(t^{\prime }\right)$ and $n_{E}\left(t^{\prime }\right)$ are AWGN noises at *D* and *E*, respectively. According to (5), the instantaneous end-to-end SNR from *S* to *D* and from *S* to *E* can be derived as

(6) $$\begin{array}{c} \psi_{S,D}\left(t^{\prime }\right)=\frac{P_{s}P_{R_{k}}|h_{S,R_{k}}{\left(t\right)|}^{2}{\left|h_{R_{k},D}\left(t^{\prime }\right)\right|}^{2}}{(P_{s}|h_{S,R_{k}}{\left(t\right)|}^{2}+P_{R_{k}}|h_{R_{k},D}\left(t^{\prime }\right){|}^{2}+\sigma^{2})\sigma^{2}},\\ \psi_{S,E}\left(t^{\prime }\right)=\frac{P_{s}P_{R_{k}}|h_{S,R_{k}}{\left(t\right)|}^{2}{\left|h_{R_{k},E}\left(t^{\prime }\right)\right|}^{2}}{(P_{s}|h_{S,R_{k}}{\left(t\right)|}^{2}+P_{R_{k}}|h_{R_{k},E}\left(t^{\prime }\right){|}^{2}+\sigma^{2})\sigma^{2}}, \end{array}$$

respectively. Thus, the end-to-end channel capacity from *S* to *D* and *S* to *E* can be given by

(7) $$\begin{array}{c} C_{S,D}\left(t^{\prime }\right)=\frac{1}{2}\log_{2}\left(1+\psi_{S,D}\left(t^{\prime }\right)\right),\\ C_{S,E}\left(t^{\prime }\right)=\frac{1}{2}\log_{2}\left(1+\psi_{S,E}\left(t^{\prime }\right)\right), \end{array}$$

respectively. The end-to-end secrecy rate from *S* to *D* is given by

(8) $$\begin{array}{c} C_{S,D}^{\left(s\right)}\left(t^{\prime }\right)={\left[\theta -C_{S,E}\left(t^{\prime }\right)\right]}^{+}, \end{array}$$

where ${\left[z\right]}^{+}=\max\left(o,z\right)$, and θ is the target rate of the two-hop AF buffer-aided relay network.

## 4. The Framework of Information Transmission Based on MDP

**Figure 2:**
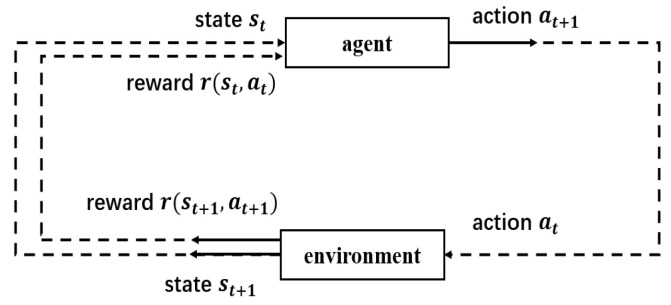
An MDP, which consists of state $s_{t}$, action $a_{t}$, policy $\pi (a_{t}|s_{t})$, reward $r(s_{t},a_{t})$, return $U_{t}$, environment and an agent.

To design a buffer-aided relay selection scheme that enables reliable and secure communications in two-hop wireless relay networks, we need to first analyze the information transmission process in two-hop wireless relay networks. Due to the Markovian property of the process of receiving and forwarding information in the buffers, the information transmission process in two-hop wireless relay networks can be modeled as an MDP to analyze. As shown in Figure 2, a complete MDP consists of a five-tuple (state $s_{t}$, action $a_{t}$, policy $\pi (a_{t}|s_{t})$, reward $r(s_{t},a_{t})$), return $U_{t}$, environment and an agent. This section describes in detail how to model the process of information transmission in two-hop wireless relay networks as an MDP.

### 4.1. Agent and Environment

In the MDP, the agent can perceive the state of the environment, take actions according to the state and adjust the decisions based on the feedback of the environment. In the two-hop AF buffer-aided relay network, the central node is regarded as the agent in the MDP and the whole two-hop AF buffer-aided relay network is modeled as the environment in the MDP. The state of the environment will be changed by action of the agent, which can be perceived by the agent. In addition, the environment will give the agent feedback after each decision made by the agent.

### 4.2. State

For the two-hop AF buffer-aided relay network, this paper defines the state space s(t) at time slot *t* as s(t)={l(t),b(t)}, where l(t) and b(t) are the link states of all links and the buffer states of all buffer queues at time slot *t*, respectively. The link states l(t) at time *t* are defined as

(9) $$\begin{array}{c} l\left(t\right)=\{l_{0,1}\left(t\right),l_{0,2}\left(t\right),\cdots ,l_{0,K}\left(t\right),l_{1,1}\left(t\right),\cdots ,l_{1,K}\left(t\right)\}, \end{array}$$

where $j=0,l_{0,k}\left(t\right)$ is the link state of *S* to the corresponding $R_{k}$ link; $j=1,l_{1,k}\left(t\right)$ is the link state of the corresponding $R_{k}$ to *D* link. As we assume that the eavesdropping node *E* only intercept signals from the $R_{k}$ to *D* link, only the reliability issue of the transmission link needs to be considered in the first hop. The value of $l_{0,k}\left(t\right)$ is taken as follows.

- $l_{0,k}\left(t\right)=0$ denotes $C_{S,R_{k}}\left(t\right)\leq \theta$ and the corresponding link is unreliable. When $l_{0,k}\left(t\right)=0$, the corresponding link can not transmit the signals at the target rate θ.
- $l_{0,k}\left(t\right)=2$ denotes $C_{S,R_{k}}\left(t\right)\geq \theta$ and the corresponding link is reliable. When $l_{0,k}\left(t\right)=2$, the corresponding link can transmit the signals at the target rate θ.

For an $R_{k}$ to *D* link, the reliability and security of the link are both considered due to eavesdropping by *E*. The value of $l_{1,k}\left(t\right)$ is taken as follows.

- $l_{1,k}\left(t\right)=0$ denotes $C_{S,D}\left(t\right)<\theta$ and the corresponding link is unreliable. When $l_{1,k}\left(t\right)=0$, the corresponding link can not transmit the signals at the target rate θ.
- $l_{1,k}\left(t\right)=1$ denotes $C_{S,D}\left(t\right)\geq \theta$, $C_{S,D}^{\left(s\right)}\left(t\right)<\zeta$ and the corresponding link is reliable but not secure, where ζ is the target secrecy rate. When $l_{1,k}\left(t\right)=1$, the corresponding link can transmit the signals with the target rate θ but cannot transmit the signals at the target secrecy rate ζ.
- $l_{1,K}\left(t\right)=2$ denotes $C_{S,D}\left(t\right)\geq \theta$, $C_{S,D}^{\left(s\right)}\left(t\right)\geq \zeta$ and corresponding link is reliable and secure. When $l_{1,k}\left(t\right)=2$, the corresponding link can transmit the signals at the target secrecy rate ζ.

Regarding one buffer-aided relay node $R_{k}$, there are two links, i.e., an *S* to $R_{k}$ link and an $R_{k}$ to *D* link, so the buffer state b(t) at time *t* are defined as

(10) $$\begin{array}{c} b\left(t\right)=\{b_{0,1}\left(t\right),\cdots ,b_{0,K}\left(t\right),b_{1,1}\left(t\right),\cdots ,b_{1,K}\left(t\right)\}, \end{array}$$

where $b_{j,k}\left(t\right)\in \left\{0,1,\cdots ,L\right\}$, j∈{0,1}, k∈{1,2,⋯,K}, because the length of buffer queue is *L*. If the selected link is an *S* to $R_{k}$ link and $b_{0,k}\left(t\right)=L$, the corresponding buffer-aided relay node $R_{k}$ is unavailable at this time because its buffer queue $Q_{k}$ is full, it can not receive the signals from *S*. If selected link is an $R_{k}$ to *D* link and $b_{1,k}\left(t\right)=0$, the corresponding buffer-aided relay node $R_{k}$ is also unavailable at this time because its buffer queue $Q_{k}$ is empty, it can not forward the signals to *D*.

According to the above analysis, we can conclude that the size of link state space l(t) and buffer state space b(t) are $6^{K}$ and ${\left(L+1\right)}^{2K}$, respectively. As s(t)={l(t),b(t)}, the size of state space s(t) is ${\left(6{\left(L+1\right)}^{2}\right)}^{K}$.

### 4.3. Action and Policy

In two-hop AF buffer-aided relay networks, the selection of a link for transmitting the signals is modeled as action in the MDP. The set of links that the agent can choose at time *t* is modeled by the action space a(t).

At state $s_{t}$, if the agent selects an *S* to $R_{k}$ link to transmit the signals, we denote $a_{t}=l_{\left(0,k\right)}$. If the agent selects an $R_{k}$ to *D* link to transmit the signals, we denote $a_{t}=l_{\left(1,k\right)}$. It is worth noting that when the states of all *S* to $R_{k}$ links are 0 and the states of all $R_{k}$ to *D* links are not equal to 2, the agent will select no link to transmit the signals (i.e., a connection outage event occurs directly) and this case is denoted as $a_{t}=\emptyset$. Based on the analysis above, we also can deduce that the size of the action space a(t) is 2K+1.

To guarantee the reliable and secure communications between legitimate users, if the link selected to transmit the signals is unreliable, then a connection outage event occurs. If the selected link is not secure, then a secrecy outage event occurs. Therefore, after the agent acts the action $a_{t}$, the environment may enter a new state $s_{t+1}$, or remain in the current state $s_{t}$ due to the connection outage or secrecy outage. In addition, if the selected link is reliable and secure but the corresponding buffer is unavailable, a connection outage event also happens. Transmission is considered successful only if the selected link is reliable and secure (for an *S* to $R_{k}$ link, the selected link is only required to be reliable) and the corresponding buffer is available. Table 2 shows the results of performing actions in different link states and buffer states.

In the MDP, the policy function $\pi (a_{t}|s_{t})$ is the probability that the agent acts action $a_{t}$ at state $s_{t}$ and is denoted by

(11) $$\begin{array}{c} \pi \left(a_{t}|s_{t}\right)=P\left(a_{t}|s_{t}\right). \end{array}$$

From (11), we can observe that $\pi (a_{t}|s_{t})$ will affect the choice of an action and also the reward for the action.

### 4.4. Reward and Return

The reward is the feedback given to the agent by the environment after the agent acts an action $a_{t}$ in a state $s_{t}$, and is noted as $r(s_{t},a_{t})$. The reward can be divided into three categories: positive reward, negative reward and neutral reward.

- Positive reward: the selected link satisfies the transmission requirements, in which the target transmission rate θ and target secrecy transmission rate ζ are both considered, and the corresponding buffer-aided relay node is available.
- Negative reward: the selected link can not satisfy the transmission requirements or the corresponding buffer-aided relay node is unavailable.
- Neutral reward: no link is selected.

In the MDP, the accumulated reward from the beginning time *t* to the end time t+n is called as the return, denoted by $U_{t}$. The expression of return $U_{t}$ is given by

(12) $$\begin{array}{c} U_{t}=r\left(s_{t},a_{t}\right)+\gamma *r\left(s_{t+1},a_{t+1}\right)+\cdots +\gamma^{n}*r\left(s_{t+n},a_{t+n}\right)=r\left(s_{t},a_{t}\right)+\gamma *U_{t+1}, \end{array}$$

where γ is the discount factor in the MDP. Moreover, the conditional expectation of the return $U_{t}$ of acting action $a_{t}$ in state $s_{t}$ is defined as the action-value function $Q_{\pi }\left(s_{t},a_{t}\right)$ and

(13) $$Q_{\pi }\left(s_{t},a_{t}\right)=E|U_{t}\left|s_{t},a_{t}\right|,s_{t}\in s\left(t\right),a_{t}\in a\left(t\right),$$

which is used to evaluate the value of state $s_{t}$ and action $a_{t}$. However, the action-value function is also influenced by the policy function π, and to eliminate the influence of the policy function π, we use the optimal action-value function $Q_{*}\left(s_{t},a_{t}\right)$ (also known as the Q-function) to evaluate the value of state $s_{t}$ and action $a_{t}$. The optimal action-value function is obtained by

(14) $$\begin{array}{c} Q_{*}\left(s_{t},a_{t}\right)=\max_{\pi }Q_{\pi }\left(s_{t},a_{t}\right),s_{t}\in s\left(t\right),a_{t}\in a\left(t\right). \end{array}$$

In the MDP, the goal of the agent is to make the return $U_{t}$ on each episode as high as possible, so the agent should select the link corresponding to the action with the maximum Q-function to transmit the signals each time.

With the above methods, we can model the process of information transmission in two-hop wireless relay networks as an MDP. Subsequently, we can use Q-learning algorithms to optimize the MDP for reliable and secure communications.

## 5. The Proposed Buffer-Aided Relay Selection Scheme

After modeling the process of information transmission as an MDP, we use Q-learning algorithms to optimize the transmission process and propose a new buffer-aided relay selection scheme based on it. Most of the existing schemes use TQL based on Q-table to optimize the transmission process and only consider reliability or security. Our proposed scheme utilizes DQL based on DNN to optimize the transmission process and considers both reliability and security. This section describes the principle of Q-learning algorithms and the steps of the proposed scheme based on DQL, respectively.

**Figure 3:**
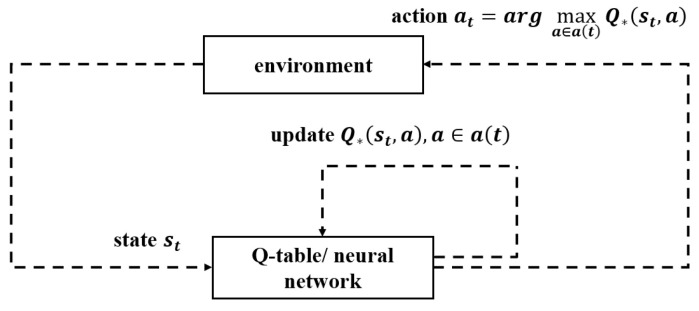
Framework of the Q-learning.

The principle of Q-learning algorithms including TQL and DQL is shown in Figure 3. The goal of the MDP is to make the return $U_{t}$ of each episode as high as possible, so the agent should perform the action with the largest Q-function value each time. In TQL, the values of Q-function are stored in Q-table and updated by the Q-learning algorithms updates Q-function by

(15) $$\begin{array}{c} Q_{*}\left(s_{t},a_{t}\right)=r\left(s_{t},a_{t}\right)+\gamma *\max_{a\in a(t+1)}Q_{*}\left(s_{t+1},a\right). \end{array}$$

In the MDP, the size of state space s(t) and action space a(t) is ${\left(6{\left(L+1\right)}^{2}\right)}^{K}$ and 2K+1, respectively. If we use the TQL based on Q-table to optimize the transmission process, it needs to occupy a lot of space to store a Q-table of ${\left(6{\left(L+1\right)}^{2}\right)}^{K}$ by 2K+1 as in Table 3 and consume a lot of time to update Q-function and search the action with the maximum Q-function. In order to reduce the space occupation and lookup time, this paper uses DQL based on DNN rather than TQL based on Q-table to optimize the transmission process. DQL uses neural network to fit the Q-function without storing the Q-function in the Q-table, so DQL can save storage space.

The proposed scheme based on DQL is divided into three phases, which are experience collection, training the network model and deploying it online. The steps of the proposed scheme are as follows.

### 5.1. Experience Collection

**Figure 4:**
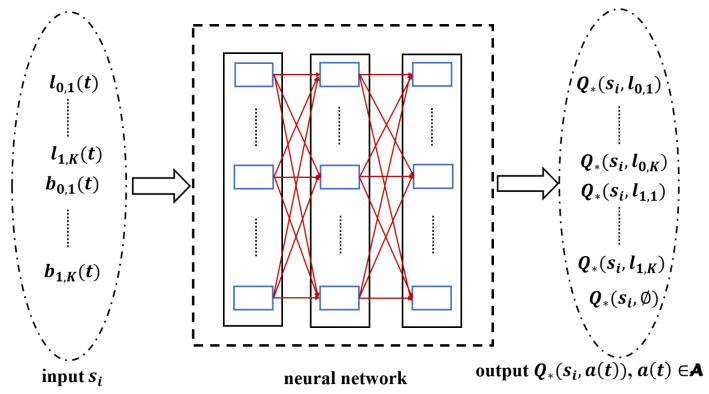
The structure of the prediction network. The input is state $s_{t}$, the output is Q-function for each action in action space a(t), $Q_{*}\left(s_{t},a\right),a\in a\left(t\right)$.

This phase focuses on collecting the experience needed to train the network model. Firstly, a DNN, which is called a prediction network, is initialized and used to fit the Q-function. The structure of the prediction network is shown in Figure 4.

The input of the prediction network is state $s_{t}$, and the output is Q-function $Q_{*}\left(s_{t},a\right)$, where a∈a(t), which corresponds to a state action at time *t*. In this phase, the ϵ-greedy policy is used to select actions to balance the exploration–exploitation dilemma. The agent chooses the action with the Q-function with 1−ϵ probability, and randomly chooses an action with ϵ probability, as shown in (16)

(16) $$a_{t}=\left\{\begin{array}{cc} \arg\max_{a\in a(t)}Q_{*}\left(s_{t},a\right), & \;prob.(1-\epsilon )\\ \mathrm{random}\;\mathrm{an}\;\mathrm{action}, & \;\;prob.\epsilon \end{array}\right.,$$

where 0<ϵ≤1. In this phase, the agent needs to explore the action space as much as possible, so ϵ is set as 1. In the training network model phase, the agent needs to train the network model by exploiting the collected experience, so as the number of training episodes increases, ϵ decreases to $\epsilon_{min}=0.1$, and the attenuation factor φ=0.998.

**Figure 5:**
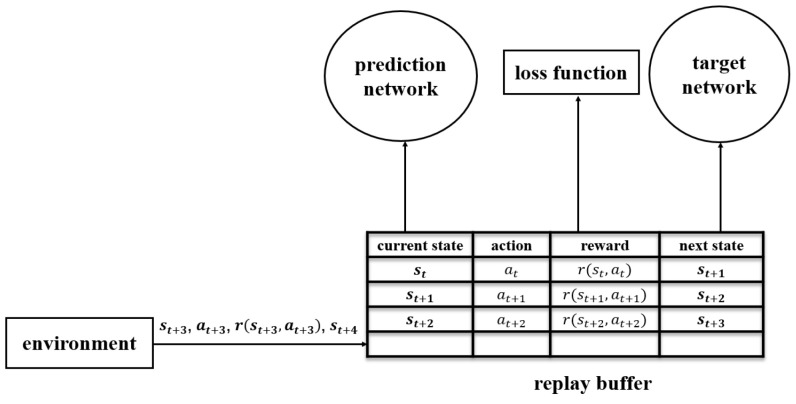
The structure of the replay buffer.

After the agent selects an action and enacts the selected action $a_{t}$, the state $s_{t}$ moves to $s_{t+1}$, and the environment returns the reward $r(s_{t},a_{t})$, a sample $\left\{s_{t},a_{t},r\left(s_{t},a_{t}\right),s_{t+1}\right\}$ is generated. The prediction network does not learn the sample immediately, but stores the sample in a buffer called as the replay buffer, which is depicted in Figure 5 and is used to store the generated experiences. The above steps of generating and collecting experience are repeated until the replay buffer is full and the prediction network starts to learn the experience.

### 5.2. Training the Network Model

**Figure 6:**
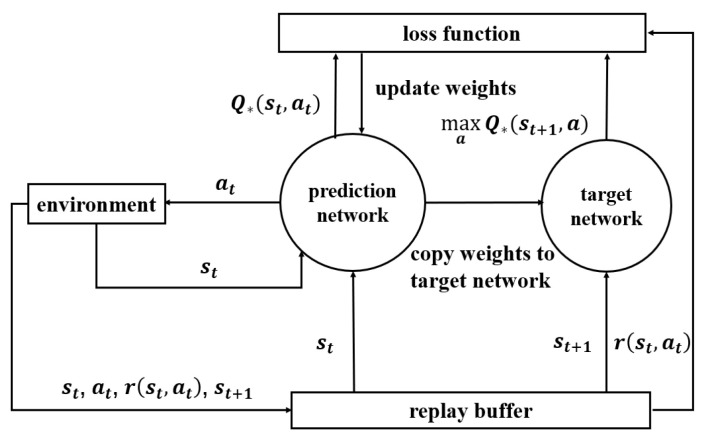
The framework of DQL with target network and experience replay.

This phase uses the experience collected in the previous phase to train and update the network model. When the replay buffer is full, the agent starts to randomly select a batch of samples for training the network model. The trick is called experience replay, which can effectively reduce the correlation between samples and improve the convergence speed of the prediction network. To avoid bootstrapping of the prediction network, this paper introduces another neural network called the target network, which has the same structure as the prediction network. The input and the output of the prediction network are $s_{t}$ and $Q_{*}\left(s_{t},a\right),a\;\in a\left(t\right)$, respectively. Similarly, the input and output of the target network are $s_{t+1}$ and $Q_{*}\left(s_{t+1},a\right),a\in a\left(t+1\right)$, respectively. As the action $a_{t}$ acts at state $s_{t}$ and the reward $r(s_{t},a_{t})$ are available according to the sample $\left\{s_{t},a_{t},r\left(s_{t},a_{t}\right),s_{t+1}\right\}$, we can obtain $Q_{*}\left(s_{t},a_{t}\right)$ and $r\left(s_{t},a_{t}\right)+\max_{a\in a(t+1)}Q_{*}\left(s_{t+1},a\right)$. Next, we calculate the error between $Q_{*}\left(s_{t},a_{t}\right)$ and $r\left(s_{t},a_{t}\right)+\max_{a\in a(t+1)}Q_{*}\left(s_{t+1},a\right)$ by using the loss function. This paper uses the mean square error (MSE) as the loss function of the prediction network and the target network. According to (16), the expression of the MSE loss function is obtained as

(17) $$\begin{array}{c} \varpi =\sum_{1}^{N}{\left(r\left(s_{t},a_{t}\right)+\gamma *\max_{a\in a(t+1)}Q_{*}\left(s_{t+1},a\right)-Q_{*}\left(s_{t},a_{t}\right)\right)}^{2}, \end{array}$$

where *N* is the batch size. Then, we update the weights of the prediction network by using the MSE loss function and copy the weights of the prediction network to the target network periodically. Finally, we repeat the above steps of learning and updating for many episodes until the prediction network and target network converge. The framework of the experience collection phase and the training network model phase of the proposed scheme is shown in Figure 6.

### 5.3. Deployment Online

After the prediction network and the target network converge, we deploy the network model online. It is worth noting that both the experience collecting and training the network model phases are offline. In this phase, the prediction network directly estimates the $Q_{*}\left(s_{t},a\right)$ corresponding to each action a,a∈a(t) based on the current state $s_{t}$, and selects the action with the maximum $Q_{*}\left(s_{t},a\right),a\in a\left(t\right)$ without training and updating the weights.

Finally, all the steps of the proposed buffer-aided relay selection scheme based on DQL are shown in Algorithm 1, where $N_{e}$ is the number of training episodes and $N_{c}$ is the capacity of the replay buffer.
**Algorithm 1** The proposed buffer-aided relay scheme based on DQL1:Initialize the environment for the two-hop AF buffer-aided relay network2:Repeat:3:**for**$\;i=1,2,\cdots ,N_{e}$  **do**4:   **for** $j=1,2,\cdots ,N_{c}$ **do**5:     (First phase: experience collection)6:     At current state $s_{t}$, select action $a_{t}$ according to ϵ-greedy policy.7:     Act the selected action $a_{t}$, and return reward $r(s_{t},a_{t})$ and next state $s_{t+1}$.8:     Generate a sample $s_{t},a_{t},r\left(s_{t},a_{t}\right),s_{t+1}$, and store it in replay buffer.9:   **end for**10:   (Second phase: training the network model)11:   Randomly select a batch of samples from replay buffer.12:   According to $s_{t}$ and $a_{t}$, get $Q_{*}\left(s_{t},a_{t}\right)$ from the prediction network.13:   According to $r(s_{t},a_{t})$ and $s_{t+1}$, get $r\left(s_{t},a_{t}\right)+\max_{a\in a(t+1)}Q_{*}\left(s_{t+1},a\right)$ from the target network.14:   Calculate the loss between $Q_{*}\left(s_{t},a_{t}\right)$ and $r\left(s_{t},a_{t}\right)+\max_{a\in a(t+1)}Q_{*}\left(s_{t+1},a\right)$ by the MSE loss function.15:   Update the weights of prediction network.16:   **if** i%100=0 **then**17:     Copy the weights of the prediction network to the target network.18:   **end if**19:**end for**20:(Third phase: deployment online)21:Deploy the prediction network online.

## 6. Simulation Results and Discussion

This section verifies the reliability and security performances of the proposed scheme by using Monte Carlo simulations, and uses the COP and SOP to measure the reliability and security performances of the proposed scheme. In the two-hop AF buffer-aided relay network, the number of buffer-aided relay nodes is K=3, the length of the buffer queue is L=3, the average channel gain is set as $\Omega_{S,R_{k}}=\Omega_{R_{k},D}=30$ dB, and $\Omega_{R_{k},E}=5$ dB. Since the power of AWGN is normalized to unity, the ratio of transmitting power to noise is set as $P_{s}/\sigma^{2}=R_{R_{k}}/\sigma^{2}=30$ dB. Furthermore, the target rate θ is set as 7 bps/Hz and the target secrecy rate ζ is set as 0.1 bps/Hz. In the proposed buffer-aided relay selection scheme based on DQL, the discount factor γ and learning rate υ of the DQL are set as 0.9 and 0.1, respectively. The capacity $N_{c}$ of replay buffer which stores samples and the batch size is set as 2000. In the phase of training the network model, the training episodes $N_{e}$ is set as 20,000, the batch size in each episode is set as 128 and the target network updates its parameters every 100 episodes. After the training of the network model is completed, the reliability and security performances of the proposed scheme are verified by 1 million Monte Carlo simulations. The lower the COP and SOP, the higher the reliability and security performances. The expressions of COP and SOP are obtained by

(18) $$\begin{array}{c} COP=\frac{n_{c}^{{}^{\prime }}}{1,000,000},\\ SOP=\frac{n_{s}^{{}^{\prime }}}{1,000,000}, \end{array}$$

where $n_{c}^{{}^{\prime }}$ and $n_{s}^{{}^{\prime }}$ are the number of connection outage events and secrecy outage events that occurred in 1 million Monte Carlo simulations, respectively.

**Figure 7:**
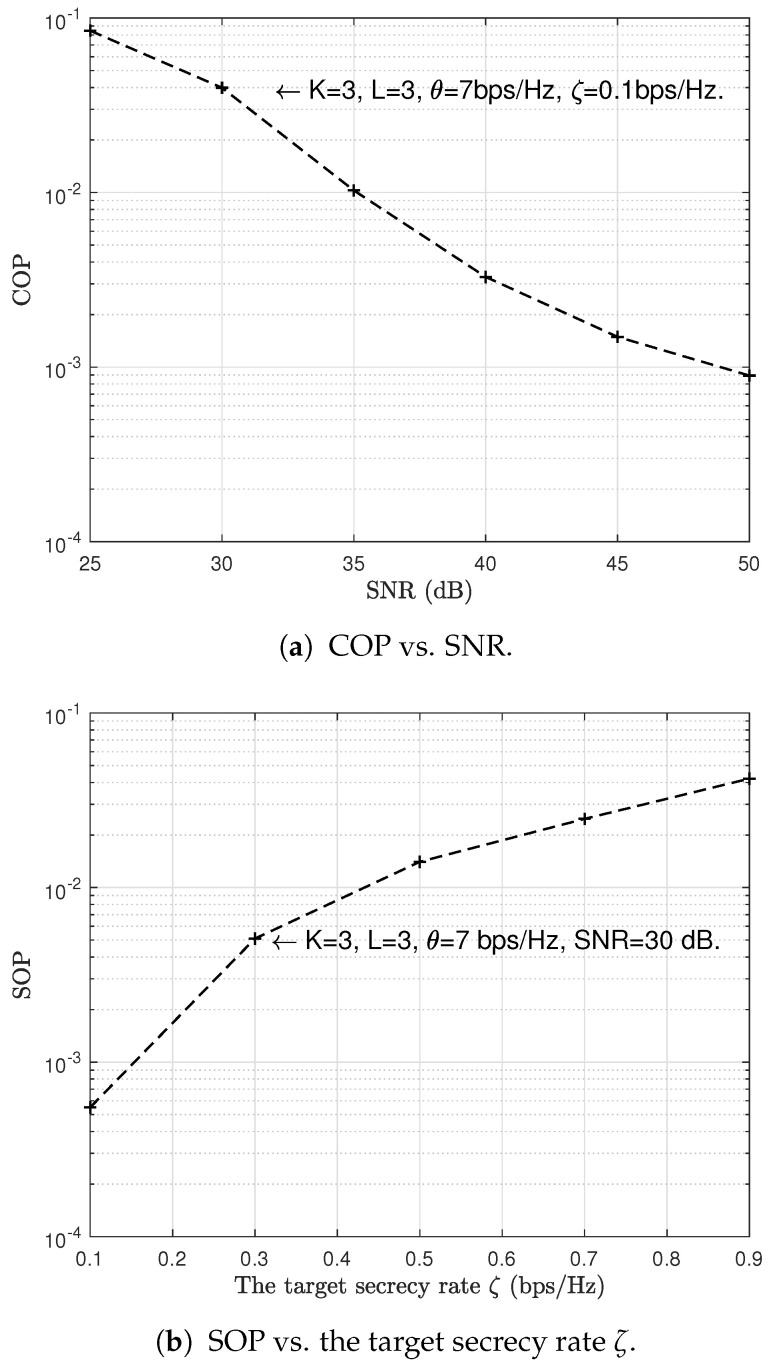
Analysis of the COP and SOP of the proposed scheme.

First, we verify the reliability and security performances of the proposed scheme. The simulation results are shown in Figure 7, where Figure 7a illustrates how the COP varies with the SNR $P/\sigma^{2}$ and Figure 7b shows how the SOP varies with the target secrecy rate ζ. From Figure 7a, we can observe that the COP decreases as the SNR increases. This is because an increase of SNR means a better transmission link and thus a lower COP. We can further see from Figure 7b that when the target secrecy rate ζ is set as 0.1 bps/Hz, the SOP can reach $10^{-4}$, and the SOP increases as the target secrecy rate ζ increases. This is because as ζ increases, fewer legitimate channels can meet the requirements to secure transmissions, which will lead to more secrecy outage events. In conclusion, the simulation results in Figure 7 confirm that our proposed scheme can achieve reliable and secure communications in two-hop wireless relay networks.

**Figure 8:**
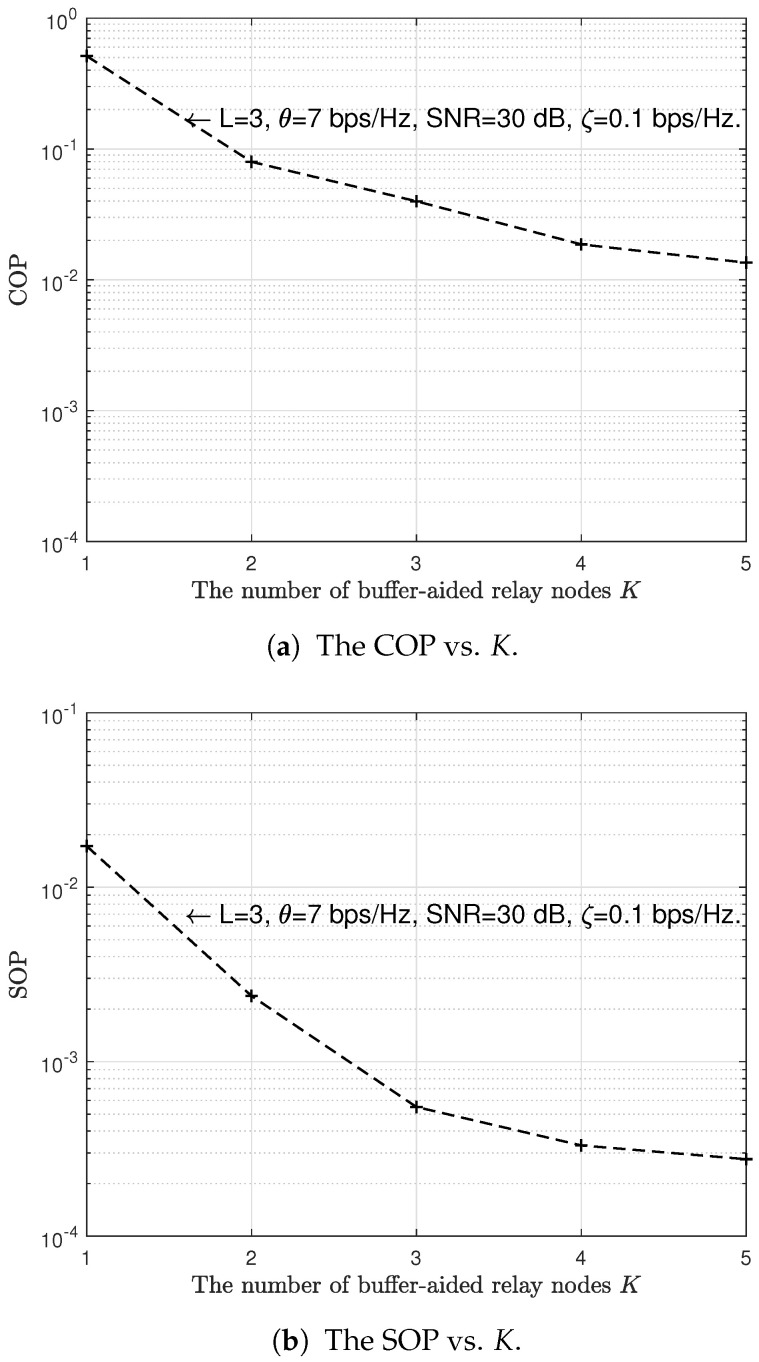
The impact of the number of buffer-aided relay nodes *K* on the COP and SOP of the proposed scheme.

**Figure 9:**
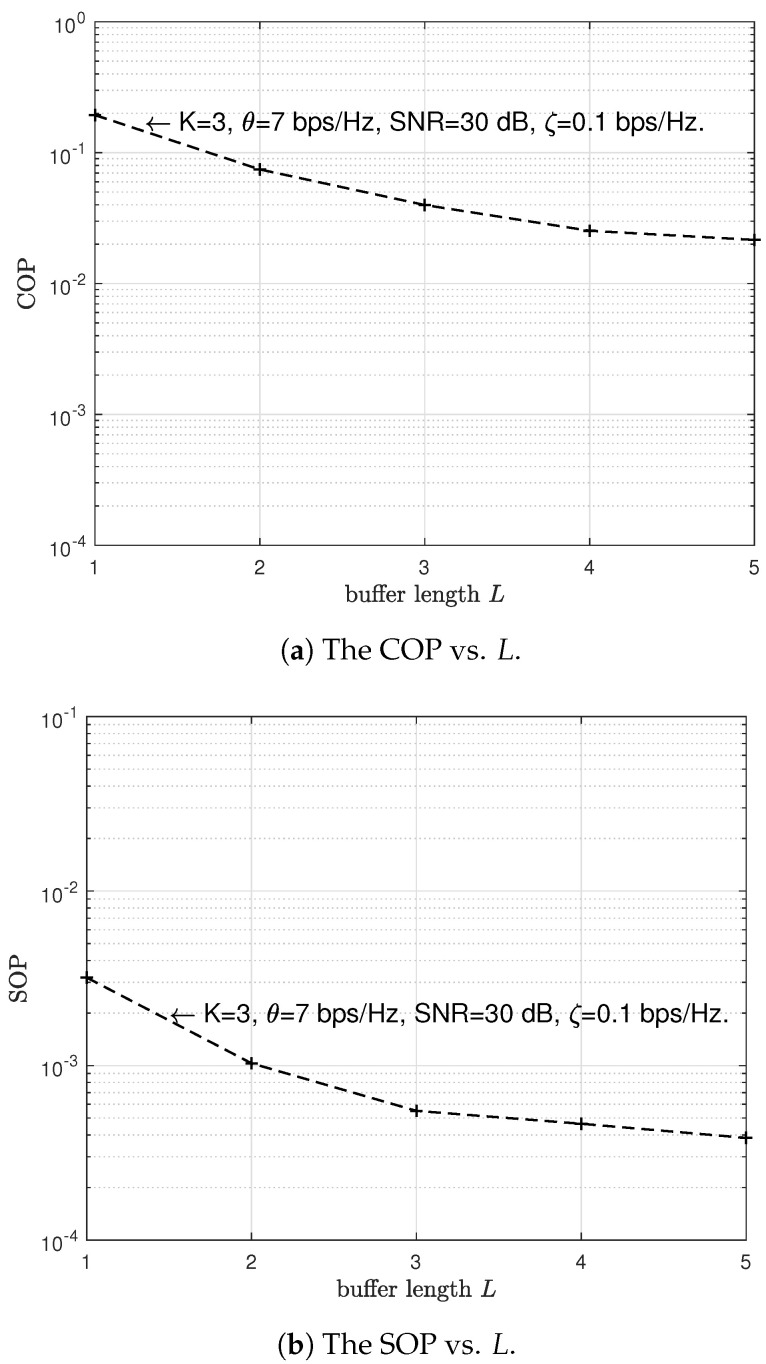
The impact of the buffer length *L* on the COP and SOP of the proposed scheme.

Then, we investigate the effect of the number of buffer-aided relay nodes *K* and the buffer length *L* on the reliability and security performances of the proposed scheme. Figure 8a and Figure 9a investigate the effect of the number of buffer-aided relay nodes *K* and the buffer length *L* on the reliability performance of the proposed scheme, respectively. In Figure 8b and Figure 9b, we, respectively, investigate the effect of the number of buffer-aided relay nodes *k* and the buffer length *L* on the security performance of the proposed scheme. We can observe from Figure 8a and Figure 9a that the COP decreases gradually as the number of buffer-aided relay nodes *K* and the buffer length *L* increase. In Figure 8b and Figure 9b, SOP also decreases as the number of buffer-aided relay nodes *K* and the buffer length *L* increase, respectively. The lower the COP and SOP, the higher the reliability and security. Resulting in Figure 8 and Figure 9 indicate that the increase in the number of buffer-aided relay nodes *K* and the buffer length *L* can improve the reliability and security performances of the proposed scheme. This is because the increase in the number of buffer-aided relay nodes *K* implies an increasing number of legitimate channels, and an increase in the buffer length *L* implies a lower probability of buffer-aided relay unavailability (the probability that a buffer is full).

**Figure 10:**
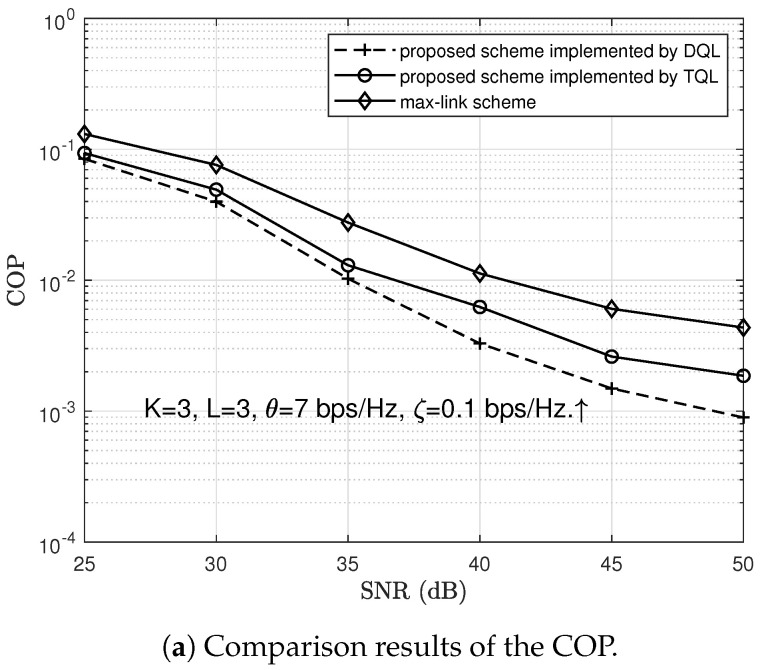

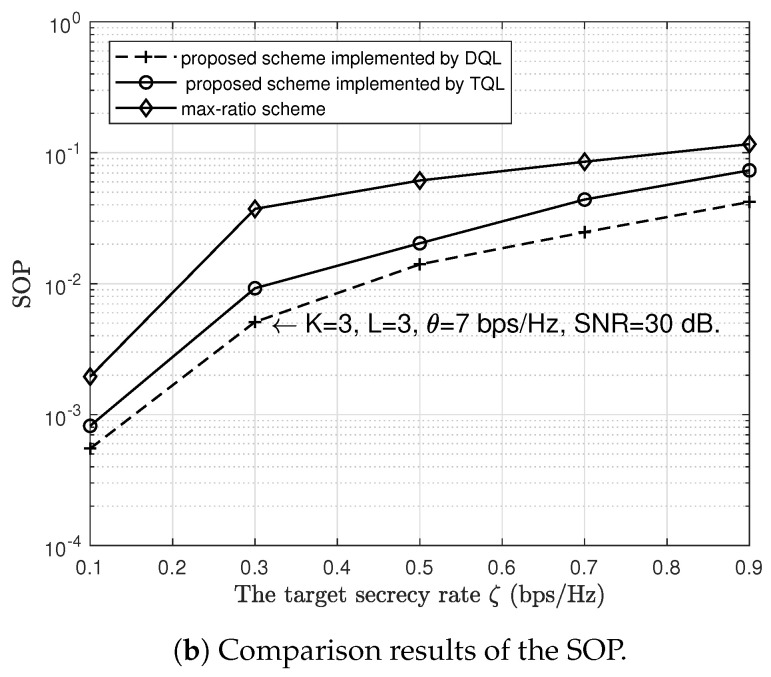
Comparison results between the proposed scheme and other schemes.

Finally, we made a comparison between our proposed scheme and two benchmark schemes (i.e., the max-link scheme and the max-ratio scheme) regarding the COP and SOP, respectively. By setting K=3, L=3, θ=7 bps/Hz, and ζ=0.1 bps/Hz, we show in Figure 10a how the COP changes by varying the SNR from 25 dB to 50 dB. The results in Figure 10a show that the COP of our scheme is always lower than that of the max-link scheme. By setting K=3, L=3, θ=7 bps/Hz, and SNR = 30 dB, we then illustrate in Figure 10b how the SOP varies by varying the target secrecy rate ζ from 0.1 to 0.9. The results in Figure 10b show that the SOP of our scheme is always lower than that of the max-ratio scheme, indicating that the security performance of the two-hop buffer-aided wireless network can be improved by adopting the DQL. In addition, we investigate the differences between the proposed scheme implemented by DQL and TQL. The comparison results are also shown in Figure 10a and Figure 10b, respectively. The comparison results clearly show that, under the same conditions, the COP and SOP of the proposed scheme implemented by DQL are both lower than those of the proposed scheme implemented by TQL.

## 7. Conclusions

This paper utilizes DQL to solve the problem of buffer-aided relay selection to achieve reliable and secure communications in a two-hop AF buffer-aided relay network with a passive eavesdropper. To propose the buffer-aided relay selection scheme, we first model the information transmission process in the network by applying an MDP. With the help of the MDP model, we then propose a novel buffer-aided relay selection scheme based on DQL to optimize the MDP. We finally verify the reliability and security performances of the proposed scheme by conducting Monte Carlo simulations and analyze how the network parameters affect the reliability and security performances of the concerned network in terms of the COP and the SOP. We also made a comparison between our proposed scheme and two benchmark buffer-aided relay selection schemes (i.e., the max-link scheme and the max-ratio scheme) regarding the COP and SOP, respectively. The results show that our proposed scheme can outperform the max-ratio scheme in terms of the SOP by 2.76 times.

## Figures and Tables

**Table 1:** The main features of our work and related works.

References and Our Work	Feature				
	System Model	Eavesdropper	Method	Reliability	Security
[19]	Two-hop DF relay network	✘	MC	**✓**	✘
[23]	Two-hop AF relay network	✘	TQL	**✓**	✘
[26]	Two-hop AF relay network	✘	MC	✘	**✓**
[40]	Delay-constrained DF relay IOT	✘	DQL	**✓**	✘
[43]	RF relay CRN	**✓** (untrusted users)	DQL+PA	**✓**	**✓**
Our Work	Two-hop AF relay network	**✓**	DQL	**✓**	**✓**

**✓** indicates that the factor is considered in the paper, and ✘ indicates that the factor is not considered in the paper.

**Table 2:** The results of performing actions in different link states and buffer states ${}^{1}$.

Action	Link State	Buffer State	Result
$l_{0,k}$	0	full	connection outage
$l_{0,k}$	0	not full	connection outage
$l_{0,k}$	2	not full	successful transmission
$l_{0,k}$	2	full	connection outage
$l_{1,k}$	2	empty	connection outage
$l_{1,k}$	2	not empty	successful transmission
$l_{1,k}$	1	empty	secrecy outage
$l_{1,k}$	1	not empty	secrecy outage
$l_{1,k}$	0	empty	connection outage
$l_{1,k}$	0	not empty	connection outage
∅	$\forall k\in \left\{1,2,\cdots ,K\right\},l_{0,k}=0,l_{1,k}\neq 2$	any	connection outage

${}^{1}$ Since TQL and DQL discussed in this paper are based on value iterations rather than policy iterations, the policy is not described in detail in this paper.

**Table 3:** The structure of the Q-table. The rows represent state space s(t) and the columns represent action space a(t).

Q-Table					
	$s_{1}$	$s_{2}$	$s_{3}$	⋯	$s_{{\left(6{\left(L+1\right)}^{2}\right)}^{K}}$
$a_{1}$	$Q_{*}\left(s_{1},a_{1}\right)$	$Q_{*}\left(s_{2},a_{1}\right)$	$Q_{*}\left(s_{3},a_{1}\right)$	⋯	$Q_{*}\left(s_{{\left(6{\left(L+1\right)}^{2}\right)}^{K}},a_{1}\right)$
$a_{2}$	$Q_{*}\left(s_{1},a_{2}\right)$	$Q_{*}\left(s_{2},a_{2}\right)$	$Q_{*}\left(s_{3},a_{2}\right)$	⋯	$Q_{*}\left(s_{{\left(6{\left(L+1\right)}^{2}\right)}^{K}},a_{2}\right)$
$a_{3}$	$Q_{*}\left(s_{1},a_{3}\right)$	$Q_{*}\left(s_{2},a_{3}\right)$	$Q_{*}\left(s_{3},a_{3}\right)$	⋯	$Q_{*}\left(s_{{\left(6{\left(L+1\right)}^{2}\right)}^{K}},a_{3}\right)$
⋮	⋮	⋮	⋮	⋮	⋮
$a_{2K+1}$	$Q_{*}\left(s_{1},a_{2K+1}\right)$	$Q_{*}\left(s_{2},a_{2K+1}\right)$	$Q_{*}\left(s_{3},a_{2K+1}\right)$	⋯	$Q_{*}\left(s_{{\left(6{\left(L+1\right)}^{2}\right)}^{K}},a_{2K+1}\right)$

## Data Availability

The data used to support the findings of this study is included within the article.

## Abbreviations

The following abbreviations are used in this manuscript:

AF	Amplify-and-forward
DQL	Deep Q-learning
COP	Connection outage probability
SOP	Secrecy outage probability
WSNs	Wireless sensor networks
CRNs	Cognitive radio networks
IoTs	Internet of Things
PLS	Physical-layer security
CSI	Channel-state information
TQL	Traditional Q-learning
MC	Markov chain
MDP	Markov decision process
FD	Full-duplex
CJ	Cooperative jamming
EH	Energy harvesting
DL	Deep learning
DNN	Deep neural network
PA	Power allocation
DF	Decode-and-forward
RF	Randomize-and-forward
HD	Half-duplex
AWGN	Additive white Gaussian noise
SNR	Signal-to-noise ratio
MSE	Mean square error

## Symbols

The following symbols are used in this manuscript:

$h_{m,n}$	Channel coefficient between *m* and *n*
$g_{m,n}$	Channel gain between *m* and *n*
Ωm,n	Average channel gain between *m* and *n*
E[.]	Expectation operator
$y_{R_{k}}\left(t\right)$	The received signal of $R_{k}$ at time *t*
$x_{S}\left(t\right)$	The signal sent by *S* at time *t*
$n_{R_{k}}\left(t\right)$	AWGN noise of $R_{k}$ at time *t*
$\psi_{S,R_{k}}\left(t\right)$	SNR of *S* to $R_{k}$ link at *t*
$C_{S,R_{K}}\left(t\right)$	Channel capacity of *S* to $R_{k}$ link
$A_{R_{k}}\left(t^{\prime }\right)$	Amplification factor of $R_{k}$ at $t^{{}^{\prime }}$
$C_{S,D}^{\left(s\right)}\left(t^{\prime }\right)$	The end-to-end secrecy rate
θ	The target rate
ζ	The target secrecy rate
$s_{t}$	The state at time *t*
$a_{t}$	The action at time *t*
s(t)	State space
a(t)	Action space
γ	Discount factor
$Q_{\pi }\left(s_{t},a_{t}\right)$	Action-value function
$Q_{*}\left(s_{t},a_{t}\right)$	The optimal action-value function
ϵ	Exploration probability
ϖ	MSE loss function
$N_{e}$	Training episodes
$N_{c}$	Capacity of replay buffer
